# Dataset for *de novo* transcriptome assembly of the African bullfrog *Pyxicephalus adspersus*

**DOI:** 10.1016/j.dib.2020.105388

**Published:** 2020-03-06

**Authors:** Naoki Yoshida, Chikara Kaito

**Affiliations:** Graduate School of Medicine, Dentistry, and Pharmaceutical Sciences, Okayama University, Tsushima-naka 1-1-1, Kita-ku, Okayama 700-8530, Japan

**Keywords:** RNA-Seq, *de novo* assembly, Transcriptome, African bullfrog, *Pyxicephalus adspersus*

## Abstract

In this article, we report the first *de novo* transcriptome assembly of the African bullfrog *Pyxicephalus adspersus*. In this data, 75,320,390 raw reads were acquired from African bullfrog mRNA using Illumina paired-end sequencing platform. *De novo* assembly resulted in a total of 136,958 unigenes. In the obtained unigenes, 30,039 open reading frames (ORFs) were detected. This dataset provides basic information for molecular level analysis of this species, which undergoes a state of dormancy under dry conditions at ordinary temperatures called estivation.

**Table:** **Specifications Table**

Subject	Biochemistry, Genetics and Molecular Biology (General)
Specific subject area	Transcriptomics
Type of data	Table, Figure
How data were acquired	Illumina HiSeq 2500 sequencing platform. The obtained data were subjected to *de novo* assembly using Trinity.
Data format	Illumina HiSeq 2500 Raw data in FASTQ format, *de novo* assembled unigene data in FASTA format
Parameters for data collection	Tissues of inner organs, intestines, muscles, and skin were collected from the young adult frog.
Description of data collection	Total RNAs isolated from 11 tissues were equivalently mixed and sequenced with Illumina HiSeq 2500 platform.
Data source location	DNA Data Bank of Japan (DDBJ) Shizuoka, Japan
Data accessibility	Data is with the article. The raw sequencing data has been deposited in DDBJ sequencing read archive (SRA): DRR164258 (https://ddbj.nig.ac.jp/DRASearch/run?acc=DRR164258). Unigene sets of the African bullfrog is also available in DDBJ/GenBank, Accession number: ICLD02000001-ICLD02136958 (http://getentry.ddbj.nig.ac.jp/) and dataset file: ICLD.gz (ftp://ftp.ddbj.nig.ac.jp/ddbj_database/tsa/).

## Value of the data

• This is the first *de novo* transcriptome assembly of the African bullfrog *Pyxicephalus adspersus*, which aestivates for 6–10 months during the hot and dry season.
• The unigene dataset will be useful resources for genomic and functional analyses of the African bullfrog and the other *Pyxicephalus* species.
• This dataset will serve as a basic information for future research to clarify differential expressed genes between active and aestivative stages of the African bullfrog.

## 1 Data description

**Fig. 1:**
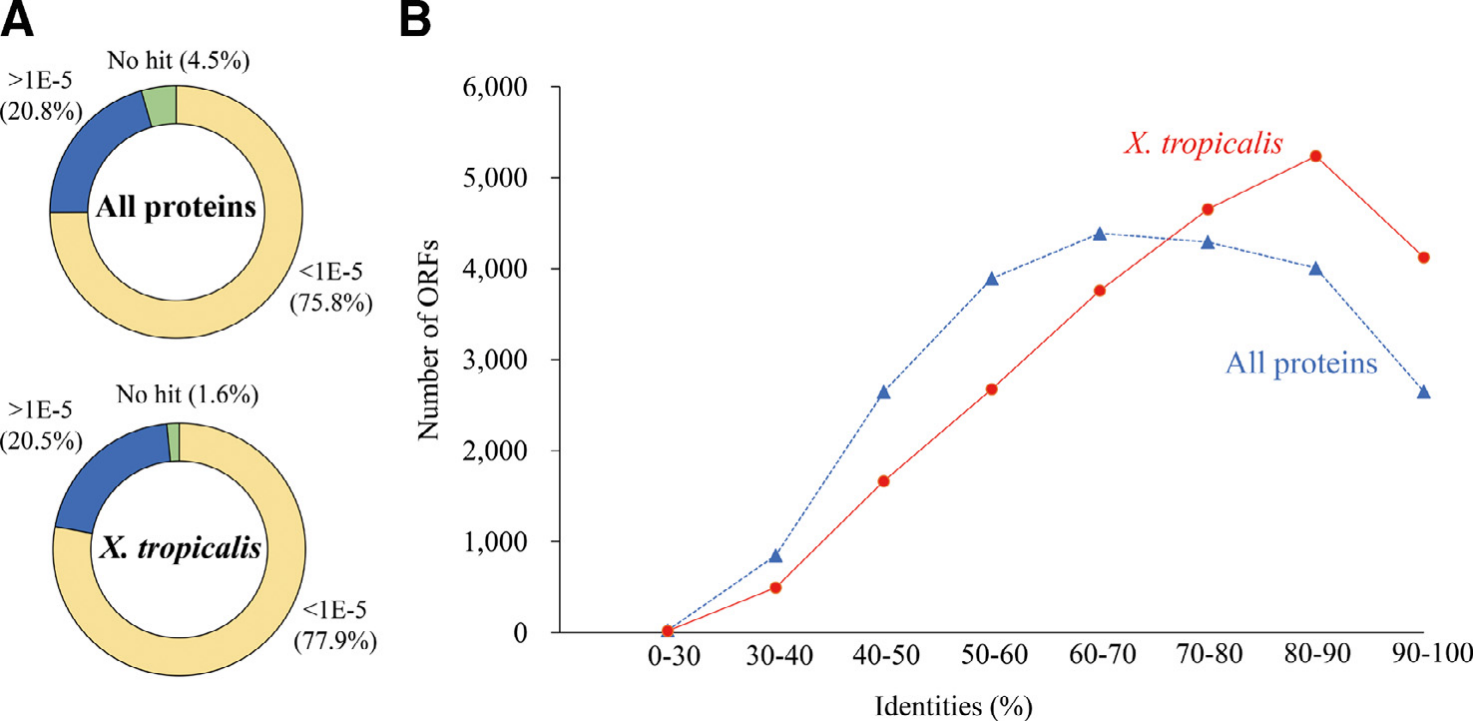
Similarity of the African bullfrog ORFs against the Uniprot protein databases. A) BLASTp search of the African bullfrog transcripts against the Uniprot database (all-proteins or *X. tropicalis* proteins), and the number of transcripts were counted according to E-values. B) Distribution of the identity of the African bullfrog ORFs with an E-value lower than 1E-05 against the Uniprot database (all-proteins or *X. tropicalis* proteins).

**Fig. 2:**
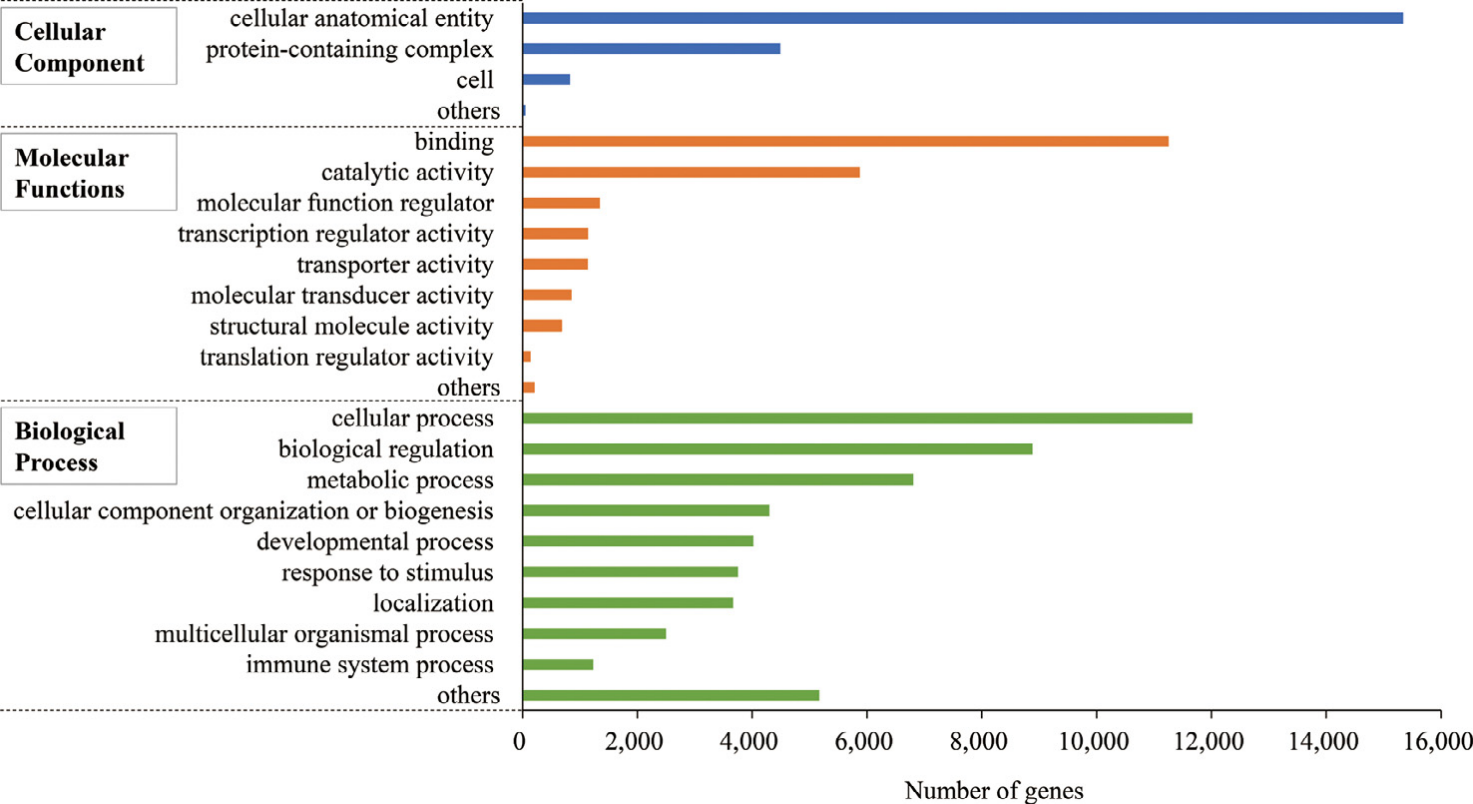
Gene ontology terms assigned to the African bullfrog ORFs. The ORFs with high identity (E-value lower than 1E-5) in a BLASTx search of the Uniprot *X. tropicalis* protein database were subjected to gene ontology analysis.

The African bullfrog, a species belonging to the family of *Pyxicephalidae*, is a large frog – body size of males is larger than 20 cm and that of females is ∼14 cm. This frog inhabits a savanna area of east Africa, south Africa, and the southern part of central Africa, where the air temperature ranges from 20–30˚C throughout the year, and there are pronounced dry and rainy seasons. During the dry season, the frogs burrow underground and form a tough cocoon to reduce evaporative water loss and decrease the respiration rate to less than 10% that during the active stage [1,2]. The estivation of the frog continues for 6–10 months. This data presents the *de novo* transcriptome assembly of the African bullfrog *Pyxicephalus adspersus*. Total RNAs were purified from 11 tissues of young male. The mixed RNA was sequenced on the Illumina HiSeq 2500 platform. The properties of the reads and the assembled sequences are shown in Table 1. The statistics of complete BUSCO hits against the tetrapoda, vertebrata, metazoan and eukaryotic databases are provided in Table 2. Supplemental table 1 refers to the sequences and annotation of the detected 30,039 ORFs. Fig. 1 shows the BLASTp homology search of the ORFs against the Uniprot protein database (all-proteins or *Xenopus tropicalis* proteins). Fig. 2 shows distribution of the ORFs on the Gene ontology (GO) analysis.

**Table 1:** Statistics of sequencing reads, transcripts and unigenes of the African bullfrog.

Sequencing statistics	Total raw reads	75,604,146
	Total clean reads	75,320,390
Assembled transcripts statistics	Total assembled transcripts	165,449
	Total assembled bases	153,613,259
Assembled unigenes statistics	Total assembled unigenes	136,958
	Total assembled bases	100,205,174
	GC %	40.7
	N50 unigene length (bp)	1428
	Mean unigene length (bp)	731
Detected ORF statistics	Total detected ORF	30,039
	N50 ORF length (aa)	587
	Mean ORF length (aa)	341
	Max ORF length (aa)	7907
	Min ORF length (aa)	100

**Table 2:** Statistics of BUSCO completeness of the assembled transcripts of the African bullfrog against the four gene sets.

BUSCO dataset				
BUSCO statistics	Tetrapoda	Vertebrata	Metazoa	Eukaryote
Total BUSCO groups	3950	2586	978	303
Complete	3410	2356	963	300
Single	2346	1701	724	226
Duplicate	1064	655	239	74
Fragment	262	140	10	3
Missing	278	90	5	0
Completeness %	86.3	91.1	98.5	99.0

## 2 Experimental design, materials, and methods

### 2.1 Ethics statement

The Animal Care and Use Committee of Okayama University approved this work (Approval number, OKU-2019300). This research was performed in strict accordance with the recommendation of the Fundamental Guidelines for Proper Conduct of Animal Experiment and Related Activities in Academic Research Institutions under the jurisdiction of the Ministry of Education, Culture, Sports, Science, and Technology in Japan, 2006.

### 2.2 Experimental animal

A captive bred African bullfrog was purchased from a specialty reptile and amphibian store (Hachurui Club, Nakano, Japan). The purchased frog was maintained in a plastic container with coarse sand (5–7 mm diameter) and water. The frog was fed every day with house crickets (*Acheta domestica*) or wax worms (the larvae of the *Galleria mellonella*) for the first 3 weeks, and then fed every day with artificial diets (Samuraijapan, Ibaraki, Japan) for 2 months.

### 2.3 Isolation of total RNA

The young adult frog (15 g body mass) was kept without feeding for 36 h. The frog was anesthetized by placing it into crushed ice for 10 min and then dissected on ice. The intestines were separated, the intestinal contents removed in phosphate buffered saline (pH7.4), and the intestines frozen in liquid nitrogen. The other tissues, including inner organs, muscle, and skin, were quickly excised to 3–5 mm^3^ (0.1–0.3 g) and frozen in liquid nitrogen. The frozen samples were maintained at -80˚C. Total RNAs were extracted from the inner organs, intestines, muscles, and skin using the chaotropic extraction protocol for mouse pancreatic RNA, described by DeLisle [3]. Frozen tissues (3–5 mm^3^, 0.1–0.3 g) were submerged in 10 ml of TRIZOL Reagent (Life Technologies, Carlsbad, CA, USA), an amount three times larger than that recommended by the supplier. The tissues were then homogenized at 14,000 rpm three times for 30 s each using the Polytron homogenizer (Kinematica AG, Luzern, Switzerland). After incubation for 5 min at room temperature, 2 ml of chloroform was added and the mixture was vortexed for 15 s. The mixture was incubated for 3 min at room temperature and centrifuged at 12,000 *g* for 10 min at 4˚C. The upper aqueous phase (4 ml) was transferred to a fresh 50-ml tube and mixed with an equivalent amount of isopropyl alcohol. The sample was centrifuged at 12,000 *g* for 10 min and an RNA pellet was obtained. The RNA pellet was vortexed with 10 ml of 75% ethanol and centrifuged at 7500 *g* for 5 min at 4 ˚C. The RNA pellet was air-dried and dissolved in 200 µl of RNase-free water by incubating for 10 min at 55 ˚C. Further purification to remove contaminated genomic DNA was performed using a Monarch Total RNA Miniprep Kit (New England Biolabs, MA, USA), according to the supplier's protocol. The RNA was eluted from a column by 100 µl of RNase-free water and kept at −80 ˚C. The concentration and purity of the isolated RNA was determined using a spectrophotometer and RNA integrity was assessed by RNA ScreenTape assay. The concentrations of RNA isolated from 11 tissue segments of inner organs, intestines, muscle, skin, and head ranged from 0.52–3.34 µg/µl or 0.38–1.60 µg/mg tissue.

### 2.4 mRNA library preparation and Illumina next-generation sequencing

An equivalent amount of total RNAs (0.52–3.34 µg/µl) isolated from 11 tissue segments of inner organs (3 segments), intestines (2 segments), muscles (2 segments), skin (3 segments), and head (1 segment) were mixed to obtain 100 ng/µl total RNA. The mixed total RNA was analyzed by TapeStation (RNA Screen tape, Agilent Technologies Ltd., USA) and determined to RIN (RNA integrity number) = 9.0. The poly (A)+ fraction was isolated from the total RNA, followed by its fragmentation. A strand-specific library with an insert size of 200 bp was prepared after conversion of the fragmented mRNA to cDNA and subjected to paired-end 2 × 100 bp sequencing on the HiSeq 2500 platform with v4 chemistry.

### 2.5 De novo transcriptome assembly and bioinformatic analysis

All analyses were performed mainly using the RNA Galaxy workbench 2.0 [4]. The 2 × 100 bp paired-end reads were checked in terms of the sequencing quality and trimmed (removal of adaptor and duplication) with quality score limit of 0.05 and a maximum number of two ambiguous nucleotides. The clean reads were then *de novo* assembled by Trinity 2.2.0 [5]. To assess the completeness of the assembled transcripts, the Benchmarking Universal Single-Copy Orthologs tool (BUSCO) was used [6]. After decreasing the isoform redundancy of the transcripts with using the CD-hit [7] and SuperTranscripts [8], unigene data set was generated. The open reading frame (ORF) in the unigenes was detected by Transdecoder [4] under the following conditions: search as Both Strand, open-ended sequence, minimum length (codons) as 100 amino acids and genetic code as standard.

### 2.6 Functional annotation

The detected ORFs were homology searched using local BLASTp (National Center for Biotechnology Information, NCBI) against the Uniprot database (https://www.uniprot.org/) (all-proteins or *Xenopus tropicalis*). Homologous proteins found in the Uniprot database (*X. tropicalis*) with an E-value lower than 1E-5 were subjected to gene ontology (GO) analysis [9] to assign the GO terms of biologic processes, molecular functions, and cellular components.

## Conflict of Interest

The authors declare that they have no known competing financial interests or personal relationships that could have appeared to influence the work reported in this paper.

## Appendix Supplementary materials

Image, application 1

Image, application 2

## References

[1] Loveridge J.P., Withers P.C. (1981). Metabolism and water balance of active and cocooned African bullfrogs *Pyxicephalus adspersus*. Physiol. Biochem. Zoo..

[2] Secor S.M. (2005). Physiological responses to feeding, fasting and estivation for anurans. J. Exp. Biol..

[3] DeLisle R.C. (2014). Isolation of pancreatic RNA. Pancreapedia.

[4] Fallmann J., Videm P., Bagnacani A., Batut B., Doyle M.A., Klingstrom T., Eggenhofer F., Stadler P.F., Backofen R., Grüning B. (2019). The RNA workbench 2.0: next generation RNA data analysis,. Nucl. Acids Res..

[5] Haas B.J., Papanicolaou A., Yassour M., Grabherr M., Blood P.D., Bowden J., Couger M.B., Eccles D., Li B., Lieber M. (2013). *De novo* transcript sequence reconstruction from RNA-seq using the Trinity platform for reference generation and analysis. Nat. Protoc..

[6] Simão F.A., Waterhouse R.M., Ioannidis P., Kriventseva E.V., Zdobnov E.M. (2015). BUSCO: assessing genome assembly and annotation completeness with single-copy orthologs. Bioinformatics.

[7] Huang Y., Niu B., Gao Y., Fu L., Li W. (2010). CD-HIT Suite: a web server for clustering and comparing biological sequences. Bioinformatics.

[8] Grabherr M.G., Haas B.J., Yassour M., Levin J.Z., Thompson D.A., Amit I., Adiconis X., Fan L., Raychowdhury R., Zeng Q., Chen Z., Mauceli E., Hacohen N., Gnirke A., Rhind N., Palma F.d., Birren B.W., Nusbaum C., Lindblad-Toh K., Friedman N., Regev A. (2011). Full-length transcriptome assembly from RNA-Seq data without a reference genome. Nat. Biotechnol..

[9] Ashburner M., Ball C.A., Blake J.A., Botstein D., Butler H., Cherry J.M., Davis A.P., Dolinski K., Dwight S.S., Eppig J.T., Harris M.A., Hill D.P., Issel-Tarver L., Kasarskis A., Lewis S., Matese J.C., Richardson J.E., Ringwald M., Rubin G.M., Sherlock G. (2000). Gene Ontology: tool for the unification of biology. Nat. Genet..

